# Prevalence and Genetic Characterization of *Chlamydia trachomatis* Isolates From Reproductive Age Women in Trachoma-Endemic and Nonendemic Areas of Tanzania

**DOI:** 10.1155/cjid/5563047

**Published:** 2025-11-25

**Authors:** Rehesina H. Senkoro, Donath Damian, Hussein M. Juma, Lucy Namkinga

**Affiliations:** ^1^Department of Microbiology, Kairuki University, Dar es Salaam, Tanzania; ^2^Department of Biochemistry and Pharmacology, Mbeya College of Health and Allied Sciences, University of Dar es Salaam, Mbeya, Tanzania; ^3^Department of Molecular Biology, College of Natural and Applied Sciences, University of Dar es Salaam, Dar es Salaam, Tanzania; ^4^Department of Pharmacology and Internal Medicine, St. Francis University College of Health and Allied Sciences, Ifakara, Tanzania

**Keywords:** *Chlamydia trachomatis*, genotypes, prevalence, Tanzania, women

## Abstract

**Background:**

*Chlamydia trachomatis* is the leading cause of sexually transmitted infections globally, responsible for various conditions including trachoma, genital infections, lymphogranuloma venereum, reactive arthritis, pneumonia, and neonatal conjunctivitis. This bacterium has 19 identified genotypes. This study aimed to evaluate the prevalence and genotype characterization of genital *Chlamydia* among women of reproductive age in both trachoma-endemic and nonendemic regions of Tanzania.

**Methods:**

From May 2022 to May 2023, we conducted a cross-sectional study involving 400 women attending antenatal and child health clinics in district hospitals located in trachoma-endemic and nonendemic areas. Endocervical swabs were collected, and DNA was extracted for analysis. Polymerase chain reaction (PCR) was used to amplify the *ompA* gene, and the resulting PCR products were sequenced and compared with reference sequences from GenBank using BLAST to identify genotypes.

**Results:**

The prevalence of genital *Chlamydia* was 2.3% (9 cases). The genotypes detected were L1 and F, each accounting for 11.1% of the cases. The infection rate was particularly high among women aged 25 years or younger.

**Conclusion:**

The study found a significant prevalence of *Chlamydia trachomatis* among young women (≤ 25 years) and identified genotypes F and L1. These results highlight the need for ongoing surveillance and targeted public health strategies to manage and prevent *Chlamydia* infections, particularly among young women in both trachoma-endemic and nonendemic regions of Tanzania.

## 1. Introduction

*Chlamydia trachomatis* is the most prevalent sexually transmitted bacterium globally, affecting approximately 129 million people each year [1]. In reproductive-age women, it poses significant risks, leading to various reproductive health complications such as ectopic pregnancy, infertility, and preterm delivery [2, 3]. In addition, *Chlamydia trachomatis* is responsible for other clinical conditions, including trachoma [4], adult conjunctivitis [5], proctitis [6], lymphogranuloma venereum (LGV) [7], reactive arthritis [8], and conjunctivitis and pneumonia in infants [9].

A critical challenge in managing *Chlamydia trachomatis* infections is that about 70% of infected women are asymptomatic, which perpetuates the spread of the infection [10, 11]. Untreated infections can lead to severe complications and increase susceptibility to HIV and human papillomavirus (HPV) [2, 12–14]. Thus, timely diagnosis and treatment are essential for controlling this public health issue.

The control of *Chlamydia trachomatis* is further complicated by its genetic diversity and broad range of tropism [15]. There are 19 known genotypes of *C. trachomatis*, each associated with different clinical presentations: Genotypes A to C are linked to trachoma, Genotypes D to K are associated with urogenital and neonatal infections, and Genotypes L1 to L3 cause LGV [16]. Notably, LGV may not always present with the typical symptoms of mucosal ulcers and intrapelvic lymphadenopathy in women. Instead, it can manifest as cervicitis with vaginal discharge or remain a persistent, asymptomatic infection [17, 18]. This poses a particular challenge in low-resource settings where syndromic management is often used [19]. For instance, LGV presenting as cervicitis with vaginal discharge might be inappropriately treated with standard regimens for vaginal discharge, whereas LGV requires a prolonged 3-week treatment regimen [20].

Characterizing *Chlamydia trachomatis* genotypes is vital for accurate diagnosis and effective treatment. While previous studies in Tanzania have reported a wide range of *Chlamydia* prevalence, from 1% to 36.2% [21–25], they have not identified the circulating genotypes. This study aims to determine the prevalence of genital *Chlamydia* and analyze the distribution of genotypes among women of reproductive age attending reproductive and child health clinics in both trachoma-endemic and nonendemic areas in Tanzania.

## 2. Methodology

### 2.1. Study Design

A cross-sectional analytical study was conducted between May 2022 and May 2023. The study targeted women of reproductive age visiting child health clinics at district hospitals located in the Dodoma Region, known for trachoma endemicity, and the Dar es Salaam Region, where trachoma is nonendemic, within Tanzania.

#### 2.1.1. Study Population

The study included sexually active women aged 18–45 years who consented to provide samples for diagnosing genital tract infections. Women were excluded if they had used antibiotics in the past 2 weeks or were menstruating at the time of the study.

#### 2.1.2. Sample Size

The sample size was determined using the Cochran formula [26], with a 95% confidence interval and a 5% margin of error, based on a reported prevalence of genital *Chlamydia* of 36.21% from Ramadhani et al. [22]. This calculation indicated a minimum sample size of 389 women. To ensure robustness, a total of 400 women were recruited for the study.

#### 2.1.3. Sampling

A two-stage sampling approach was employed. Two districts were randomly selected from each region, and district hospitals in these districts were chosen for the study. Eligible women attending reproductive and child health clinics at these hospitals were enrolled consecutively after providing written consent.

#### 2.1.4. Collection

Data were collected using a pretested structured questionnaire in Swahili, which gathered information on sociodemographic details, behavioral factors, and symptoms. Endocervical and vaginal specimens were collected using Dacron swabs for diagnosing reproductive tract infections.

### 2.2. Laboratory Procedures

#### 2.2.1. DNA Extraction and Polymerase Chain Reaction (PCR) Amplification

DNA extraction was performed according to the ZymoBIOMICS Kit instructions, and the extracted DNA was stored at −20°C. For PCR amplification, the reaction mixture comprised 2 μL of DNA template, 8 μL of sterile water, 12.5 μL of Master Mix, and 1.25 μL of each primer, totaling 25 μL. Amplification of the outer membrane protein A (*ompA*) gene was carried out following the protocols of Kiguen et al. [27] and Hussein et al. [28]. Primers NRO (5′CTCAACTGTAACTGCGTATTT3′) and NLO (5′ATGAAAAAACTCTTGAAATCG3′) were used to amplify a 1087-bp segment of the *ompA* gene. The PCR conditions included an initial denaturation at 95°C for 4 min, followed by 49 cycles of denaturation at 95°C for 1 min, annealing at 55°C for 1 min, and elongation at 72°C for 1.5 min.

For detecting the cryptic plasmid, a 201-bp fragment of C. *trachomatis* was amplified using primers CTP2 (5′-TTCCCCTTGTAATTCGTTGC-3′) and CTP1 (5′-TAGTAACTGCCAClTCATCA-3′). The amplification protocol started with an initial denaturation at 95°C for 4 min, followed by 35 cycles of denaturation at 95°C for 1 min, annealing at 55°C for 1 min, elongation at 72°C for 1.5 min, and a final elongation at 72°C for 4 min [29]. PCR products were visualized under UV light after electrophoresis on a 1% agarose gel with SYBR Green DNA staining. Positive control (Amplirun *Chlamydia* DNA control VC MBC012) and negative control (nuclear-free water) were included in each PCR run to serve as quality control for the reaction. Positive samples were sent to Macrogen for sequencing. LGV was detected by sequencing *Chlamydia trachomatis* DNA using next generation sequencing (NGS). Targeted sequencing focuses on the major *ompA* gene and the polymorphic membrane protein H (*pmpH*). Nested PCR was used to amplify these target gene regions from the DNA of a clinical sample. The resulting sequences were compared against reference sequences for known *C. trachomatis* genovars (D-K and L1-L3) to determine the specific genotype present. Sequencing quality control was performed using tools such as Chromas and FinchTV for chromatogram visualization, with manual inspection to confirm peak quality and base calls; sequences with ambiguous or low-quality reads were resequenced or excluded. Coverage depth was monitored to ensure a minimum of 30x per sequence. The reference panel consisted of curated *ompA* gene sequences from GenBank (listed in Table 1), representing diverse strains and geographic origins for robust comparative analysis.

### 2.3. Sequencing Analysis

The sequences obtained were aligned with those from GenBank using MAFFT V. 7 (https://mafft.cbrc.jp/alignment/server/) and manually adjusted with AliView [30]. The *ompA* gene sequences were compared with reference sequences listed in Table 1. The best DNA evolution model was selected using the Akaike information criterion in MrModeltest 2.3 [31], with the F81 + I + G model applied for the first and second codons and the GTR + G model for the third codon. Bayesian inference was conducted using MrBayes 3.2.6, with posterior probabilities (PPs) used to estimate branch support [32]. Four Markov chains were run twice for a total of 10 million generations, with trees sampled every 100 generations and 25% discarded as burn-in [33]. Maximum likelihood (ML) estimates were calculated using the GTR + G + I substitution model [34] with RAxML V.8.2.10, and branch support was determined using ML bootstrapping (MLb) with 1000 replicates [35]. Statistically significant branch support was defined as Bayesian PPs ≥ 0.95 [36] and MLb values  > 70%.

### 2.4. Statistical Analysis

Statistical analysis was carried out using SPSS Version 23. Descriptive statistics were computed for sociodemographic data, with categorical variables expressed as percentages and continuous variables summarized as means with standard deviations. The prevalence of *Chlamydia* was calculated as the proportion of participants with positive test results. Associations between sociodemographic factors and *Chlamydia* prevalence were examined using chi-square and Fisher's exact tests. A logistic regression model was used to identify independent factors associated with genital *Chlamydia*. The dependent variable was *Chlamydia* infection status (positive vs. negative). Independent variables that were entered in the multivariable logistic regression model were residence, age group, education level, employment, and marital status. Adjusted odds ratios and 95% confidence intervals were computed, with a *p* value < 0.05 considered statistically significant. Adjusted odds ratios reflect the association between each listed predictor variable and *Chlamydia* infection (outcome), controlling for all other variables in the model.

### 2.5. Ethical Approval

The study protocol received approval from the National Health Research Ethics Committee (NatHREC) in Dar es Salaam, Tanzania, under approval number NIMR/HQ/R.8a/Vol. IX 3720. Permissions were also obtained from the Regional Medical Officer, District Medical Officer, and the hospital director. Written informed consent was obtained from all participants prior to enrollment, and data confidentiality was maintained by storing data on a password-protected computer by the principal investigator.

## 3. Results

### 3.1. Demographic Characteristics of the Study Population

A total of 400 women from both trachoma-endemic and nonendemic areas participated in the study. The average age of the participants was 28 years, with a standard deviation of 6.8 years. Among them, 51.0% resided in areas where trachoma is not endemic. Most of the participants (56.3%) were older than 25 years. Regarding educational attainment, 51.75% had secondary or postsecondary education, and 66.3% were employed. The distribution of marital status included 41.0% married, 39.5% single, and 19.5% cohabiting (Table 2).

### 3.2. Prevalence of Genital *Chlamydia trachomatis*

Out of 400 samples tested, 9 were positive for *C. trachomatis* DNA, yielding a prevalence rate of 2.3% (95% CI: 2.27–2.33). The prevalence was higher in trachoma-endemic areas (1.8%) compared to nonendemic areas, although this difference was not statistically significant. *Chlamydia* prevalence was notably higher among women aged 25 years or younger (4.6%) than in those older than 25 years, with this difference being statistically significant (*p*=0.02). Women with secondary and postsecondary education had a higher prevalence of *Chlamydia* (2.4%) compared to those with primary or no formal education, but this difference was not statistically significant. Employed women exhibited a lower *Chlamydia* prevalence (1.9%) compared to unemployed women, though this difference was also not statistically significant. In addition, single women had a higher prevalence of *Chlamydia* (3.2%) compared to married and cohabiting women, but this difference did not reach statistical significance (Table 3).

### 3.3. Independent Factors for Genital *Chlamydia*

In multivariable analysis, women residing in a nonendemic region for trachoma were less likely to test positive for *Chlamydia* compared to women residing in a trachoma-endemic region (AOR = 0.2; 95% CI: 0.008–0.9). Women aged over 25 years had a decreased likelihood of testing positive for *Chlamydia* compared to women aged ≤ 25 years (AOR = 0.1; 95% CI: 0.01–0.71). Marital status, education level, and employment were not independent factors for *Chlamydia* (Table 3).

### 3.4. *Chlamydia* Genotype Distribution

**Figure 1:**
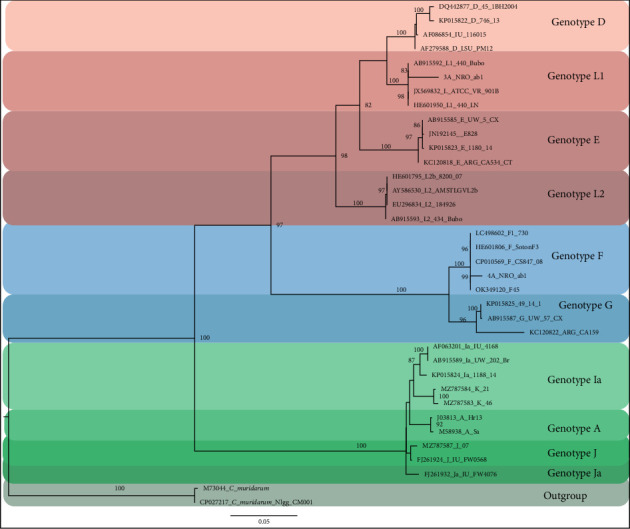
Phylogenetic tree for *ompA* gene nucleotide sequences.

Sequencing of all nine *ompA* gene PCR amplicons identified Genotypes *F* (11.1%) and L1 (11.1%). Genotype L1 was primarily found in the trachoma-endemic area, while Genotype F was identified in the nonendemic area. Phylogenetic analysis demonstrated that all sequences were closely related, indicating high genetic similarity among the strains. The *ompA* gene sequences were highly homologous, sharing over 98% similarity. The phylogenetic tree illustrated three main clusters: the first cluster included Genotypes L1, L2, D, and E; the second cluster consisted of Genotypes F and G; and the third cluster included Genotypes H, Ia, I, J, Ja, A, and K (Figure 1). Identified genotypes were deposited in the GenBank, where the accession number PV505829 was obtained, shown on the phylogenetic tree as 3A_ NRO_ab1, corresponding to Genotype L1. The accession number PV505830 is indicated on the phylogenetic tree as 4A_NRO_ab1, corresponding to Genotype F (Figure 1).

Figure 1: This phylogenetic tree illustrates the relationships of the *ompA* sequence among *Chlamydia* genotypes from both this study and GenBank, analyzed using Bayesian and ML methods. The tree is rooted with two species of *Chlamydia muridarum*. Each internal branch is accompanied by two support values: Bayesian PPs and MLbs. Branches shown in bold represent support levels of PP ≥ 0.95 and MLbs ≥ 70%. An asterisk on a bold branch indicates a node with PP = 1.0 and MLbs = 100. Branches marked with a double-slash have been truncated for clarity. Clade names follow standard naming conventions.

## 4. Discussion

This study aimed to evaluate the prevalence of genital *Chlamydia* and the distribution of genotypes among women of reproductive age in both trachoma-endemic and nonendemic areas.

### 4.1. Prevalence of Genital *Chlamydia*

Our study observed a relatively low prevalence of genital *Chlamydia* at 2.3%. The average age of the participants was 28 years, which could be a factor in this lower prevalence. This result is consistent with the range of genital *Chlamydia* prevalence reported in Tanzania, which varies from 1% to 36.2% [22–25, 37]. Furthermore, it aligns with other prevalence studies conducted among women of reproductive age in Tanzania: 1.7% [38], 3.0% [39], and 2.6% [25]. Internationally, our findings are comparable to those reported in the USA (2.0%) [40], Hong Kong (2.0%) [41], Iraq (2.6%) [42], Belgium (1.5%) [43], and Nigeria (1%) [44].

In contrast, higher prevalence rates have been reported in certain regions of Tanzania, such as 12% [45], 14% [37], and 36.2% [22]. Differences in diagnostic methods and populations studied may account for these variations. For example, these studies often focused on high-risk groups such as STI clinic attendees [37], individuals working in recreational areas [45], and infertile women [22]. Infertile women, as identified by Passos et al. [46], are a high-risk group with potentially higher infection rates than the general population [47].

Other countries have also reported higher prevalence rates among women of reproductive age, including Kenya (7.5%) [48], Nigeria (26%) [49], Ethiopia (18.9%) [50], and Uganda (26.5%) [51]. These differences may be attributed to variations in risk factors, diagnostic methods, and the effectiveness of STI prevention programs.

### 4.2. Association of Genital *Chlamydia* With Sociodemographic Factors

The study identified younger age (≤ 25) as an independent predictor of genital *Chlamydia*, consistent with other reports linking *C. trachomatis* infection predominantly to younger individuals [52]. Similar findings have been reported in Kenya [48], Uganda [53], Fiji Island [54], Iran [55], China [41], Brazil [56], and Ethiopia [50]. Younger adults engage in more sexual activity and often have multiple partners, which increases their risk of *Chlamydia* infection [57]. In addition, the squamocolumnar junction in younger women is more exposed and susceptible to infection [58].

Conversely, some studies have reported higher *Chlamydia* prevalence among women older than 25 years [42, 49, 59, 60]. Other factors such as access to preventive healthcare services or economic status may also influence *Chlamydia* prevalence [61–63]. Despite the lack of association between education level and *Chlamydia* infection in this study, targeting young women with sex education interventions could help reduce *Chlamydia* prevalence and associated morbidity.

### 4.3. Genital *Chlamydia* Genotype Distribution

The study identified Genotypes F and L1. Most published data indicate that E, F, and D are the most prevalent genotypes in genital infections [64]. Our findings are consistent with studies that have identified Genotype F as predominant in genital infections [56, 65–67].

Our study found LGV genotype L1. LGV caused by Genotypes L1, L2, and L3 is uncommon but endemic in parts of Africa, Southeast Asia, India, the Caribbean, and South America [7]. Our results are similar to findings from Mexico [68, 69] and Argentina [27, 70], where LGV genotypes have been identified. While LGV is known to affect both sexes equally, it often remains asymptomatic in women until complications develop [18]. Unlike genotypes D-K, LGV genotypes can cause systemic infections and various complications [7].

The identification of LGV genotype L1 in this study highlights a public health concern, particularly given the lack of etiologic laboratory tests for *Chlamydia* in many developing countries, including Tanzania. Tanzania employs a syndromic approach for STI management, which may not adequately address LGV. Current treatment regimens for vaginal discharge may not effectively treat LGV, which requires a prolonged 21-day course [20]. Thus, there is a need for diagnostic tests and characterization of *Chlamydia* isolates to guide appropriate treatment.

### 4.4. Phylogenetic Analysis

Phylogenetic analysis revealed three main clusters: the C-complex (Serovars A, C, H, I, Ia, J, K, and L3), the intermediate complex (Serovars F and G), and the B-complex (Serovars B, Ba, D, E, L1, and L2). The *ompA* gene tree (Figure 1) is consistent with other published *ompA* trees [27, 70, 71]. These groupings do not show a clear relationship with clinical characteristics or tissue tropism, suggesting that MOMP variability is influenced by antigenicity and immune selection [70].

### 4.5. Study Limitations

Despite its limitations, this study successfully identified women at risk of genital *Chlamydia* infections and the predominant genotypes, contributing valuable data to Tanzania's STI surveillance efforts. However, since the isolates were from women only, the general distribution of genotypes in the broader community remains unclear. Future research should include both men and women to provide a comprehensive understanding of *Chlamydia* genotype distribution. In addition, the study did not explore factors beyond demographics that may predispose individuals to *Chlamydia* infection. Nevertheless, the information on genotypes may aid in the development of vaccines and microbicides, and the prevalence data support the need for etiologic screening and early diagnosis for sexually active women under 25.

## 5. Conclusion

This study highlights that genital *Chlamydia* is prevalent among young women and identifies two circulating genotypes. Prevention programs should focus on young women to curb the spread of genital *Chlamydia*. Further research is needed on the distribution of Genotype L1 in the general population, given its asymptomatic nature in women and the inadequacy of current syndromic management regimens in treating LGV.

## Figures and Tables

**Table 1:** Reference strains of *C. trachomatis* obtained from GenBank.

Accession number	Country
D (DQ442877)	Brazil
D (AB915583)	Japan
D (X62,919)	France
D (KPO15822)	USA
D (EU191085)	Argentina
E (KC1208185)	Argentina
E (AB915585)	Japan
E (AY464144)	Australia
E (JN192145)	France
B (DQ64297)	USA
B (M33636).	UK
B (AY46914)	Australia
Ia (KP015824)	India
B (AY46914)	Australia
L2 (CP002682)	USA
L2 (AB915593)	Japan
L2 (DQ217607)	Portuguese
G (KC120822)	Argentina
G (AB915587)	Japan
G (AY464159)	Australia
G (KP015825)	India
F (CP006678)	USA
F (AB915586)	Japan
F (EU339316)	China
F (DQ442881)	Brazil
Ia (AF063201)	USA

**Table 2:** Demographic characteristics of the study population.

Characteristic	Value
Total participants	400
Average age	28 years
Standard deviation	6.8 years
Area of residence	
Nonendemic areas	51.0%
Age distribution	
Older than 25 years	56.3%
Educational attainment	
Secondary or postsecondary education	51.75%
Employment status	
Employed	66.3%
Marital status	
Married	41.0%
Single	39.5%
Cohabiting	19.5%

**Table 3:** Association between sociodemographic factors and genital *Chlamydia* among childbearing age women attending maternal and child health clinics in Tanzania.

Variables	Genital *Chlamydia*					
	N 400	Ct positive *n* (%)	COR 95% CI	*p* value	AOR 95% CI	*p* value
*Residence*						
*Chlamydia* endemic region	196	7 (3.6)	1			
Nonendemic region	204	2 (0.9)	0.3 (0.05–013)	0.01	0.2 (0.008–0.9)	0.04^∗^

*Age group*						
≤ 25 years	175	8 (4.5)	1			
> 25 years	225	1 (0.4)	0.1 (0.01–0.7)	0.01	0.2 (0.01–0.71)	0.02^∗^

*Education level*						
Secondary and above	193	4 (2.0)	1			
No education and primary	207	5 (2.4)	1.2 (0.3–4.4)	0.81	3.5 (0.48–24.8)	0.22

*Employment status*						
Not employed	133	4 (3.0)	1			
Employed	267	5 (1.9)	0.6 (0.16–2.3)	0.48	0.46 (0.06–3)	0.43

*Marital status*						
Single	158	5 (3.2)	1			
Married	164	3 (1.8)	0.6 (0.13–2.4)	0.62	0.5 (0.02–12.2)	0.73
Cohabiting	78	1 (1.3)	0.9 (0.04–3.4)	0.45	0.86 (0.06–3.2)	0.43

Abbreviations: AOR, adjusted odds ratio; CI, confidence interval; COR, crude odds ratio.

^∗^Independent factor for genital *Chlamydia*.

## Data Availability

The data that support the findings of this study are available within the article. Additional data that were generated or analyzed during the study are available from the corresponding author upon reasonable request.
